# Mating strategies with genomic information reduce rates of inbreeding in animal breeding schemes without compromising genetic gain

**DOI:** 10.1017/S1751731116001786

**Published:** 2016-08-17

**Authors:** H. Liu, M. Henryon, A. C. Sørensen

**Affiliations:** 1 Department of Molecular Biology and Genetics, Center for Quantitative Genetics and Genomics, Aarhus University, PO Box 50, 8830 Tjele, Denmark; 2 Seges, Danish Pig Research Centre, Axeltorv 3, 1609 Copenhagen V, Denmark; 3 School of Animal Biology, University of Western Australia, 35 Stirling Highway, Crawley, WA 6009, Australia

**Keywords:** mating strategies, genomic selection, genetic gain, inbreeding, genetic contributions

## Abstract

We tested the hypothesis that mating strategies with genomic information realise lower rates of inbreeding (*∆F*) than with pedigree information without compromising rates of genetic gain (*∆G*). We used stochastic simulation to compare *∆F* and *∆G* realised by two mating strategies with pedigree and genomic information in five breeding schemes. The two mating strategies were minimum-coancestry mating (MC) and minimising the covariance between ancestral genetic contributions (MCAC). We also simulated random mating (RAND) as a reference point. Generations were discrete. Animals were truncation-selected for a single trait that was controlled by 2000 quantitative trait loci, and the trait was observed for all selection candidates before selection. The criterion for selection was genomic-breeding values predicted by a ridge-regression model. Our results showed that MC and MCAC with genomic information realised 6% to 22% less *∆F* than MC and MCAC with pedigree information without compromising *∆G* across breeding schemes. MC and MCAC realised similar *∆F* and *∆G*. In turn, MC and MCAC with genomic information realised 28% to 44% less *∆F* and up to 14% higher *∆G* than RAND. These results indicated that MC and MCAC with genomic information are more effective than with pedigree information in controlling rates of inbreeding. This implies that genomic information should be applied to more than just prediction of breeding values in breeding schemes with truncation selection.

## Implications

We showed that mating strategies (minimum-MC and mating by minimising covariance between ancestral contributions) with genomic information realise lower rates of inbreeding than with pedigree information without compromising rates of genetic gain when performing truncation selection on predicted genomic-breeding values. Application of these mating strategies in animal breeding schemes is feasible in practice. They simply use the same information as for genomic prediction to pair the parents more appropriately without any extra cost and logistic constraints, which is an improvement on pedigree information. This implies that in animal breeding schemes with truncation selection, genomic data should be applied to more than just prediction of breeding values.

## Introduction

Choosing appropriate mating strategies in animal breeding reduces rates of inbreeding without compromising rates of genetic gain (Caballero *et al*., 1996). They do so by distributing genetic contributions of ancestors more evenly across mating pairs, which improves the genetic structures of breeding populations (Sonesson and Meuwissen, 2000). Two mating strategies that were developed for breeding schemes using phenotypic and pedigree data without genomic information are minimum-coancestry mating (MC) and mating by minimising the covariance between ancestral contributions (MCAC) (Wright, 1921; Henryon *et al*., 2009). These strategies are generally recommended to realise lower rates of inbreeding in breeding schemes without genomic information (Caballero *et al*., 1996; Meuwissen, 2007; Henryon *et al*., 2009; Nirea *et al*., 2012). The lower rates of inbreeding realised by MC and MCAC can be explained using the theory of long-term genetic contributions (Woolliams and Thompson, 1994; Grundy *et al*., 1998). The theory proposes that the minimum rate of inbreeding, given a pre-defined rate of genetic gain, is realised when the long-term genetic contributions of the ancestors stabilise to an exact threshold-linear relationship with their Mendelian-sampling term: genetic contributions of ancestors are zero below a threshold Mendelian-sampling term and increase linearly with the value of Mendelian-sampling terms above the threshold (Lindgren and Matheson, 1986; Grundy *et al*., 1998; Woolliams *et al*., 2002, Woolliams, 2006 and 2007). MC and MCAC realise low rates of inbreeding by increasing the independence and reducing confounding between genetic contributions of ancestors. They disperse the contributions within breeding populations and increase the number of ancestors that contribute to each descendent (Woolliams *et al*., 2002; Sørensen *et al*., 2005; Henryon *et al*., 2014). This enables selection to align the ancestors closer to the exact threshold-linear relationship and reduce rates of inbreeding (Woolliams and Thompson, 1994; Woolliams *et al*., 2002). The challenge in breeding schemes without genomic information is that there is a limit to the level of independence that can be achieved by MC and MCAC with pedigree information. Therefore, if we are to further reduce rates of inbreeding with MC and MCAC, we need information that enables us to make ancestral genetic contributions more independent so that genetic contributions can be more dispersed across the population.

Genomic information may enable MC and MCAC to disperse genetic contributions within breeding populations more effectively than pedigree information. One way to achieve this is by replacing the pedigree-relationship matrices in MC and MCAC with genomic-relationship matrices (Sun *et al*., 2013; Henryon *et al*., 2014). Genomic-relationship matrices provide more accurate estimates of relationships between individuals by tracing Mendelian segregation of chromosome segments (Hayes *et al*., 2009). This should further increase the independence and dispersion of genetic contributions within breeding populations, enabling selection to align the ancestors even closer to the exact threshold-linear relationship and bringing about further reductions in rates of inbreeding. Based on this background information, we reasoned that MC and MCAC with genomic information realises lower rates of inbreeding than MC and MCAC with pedigree information without compromising rates of genetic gain. We tested this hypothesis by stochastic simulation. We simulated MC and MCAC in five breeding schemes with different family structures and heritabilities. We measured inbreeding as homozygosity due to identity-by-descent. Genetic gain was measured as increases in true breeding values.

## Material and methods

### Experimental design

We used stochastic simulation to compare rates of inbreeding and genetic gain realised by MC and MCAC with pedigree and genomic information in five breeding schemes. We also simulated random mating (RAND) as a reference point. Generations were discrete, animals were truncation-selected for a single trait that was controlled by 2000 quantitative trait loci (QTL), and the trait was observed for all selection candidates before selection. The criterion for selection was genomic-breeding values predicted by a ridge-regression model using 8257 markers. The five breeding schemes differed for mating ratio (1, 2 or 6 dams/sire), litter size (10, 20 or 60 offspring/litter) and heritability of the trait (0.1 or 0.4). Each combination of breeding scheme and mating strategy was run for 20 discrete generations and replicated 100 times.

### Mating strategies

#### Minimum-coancestry mating

Selected sires and dams were paired by minimising the average coancestry of the proposed matings. Pedigree-additive relationship ***A*** or the genomic relationship ***G*** were created after (Meuwissen and Luo, 1992) and (Yang *et al*., 2010). The dimensions of ***A*** and ***G*** were *N*_s_×*N*_d_, where *N*_s_ and *N*_d_ are the number of selected sires and dams, and the elements 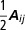 and 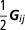 are expected coefficients of coancestry of individuals *i* and *j*. These coefficients are equivalent to the inbreeding coefficients of their offspring. Minimum-MC were performed using the algorithm described in Henryon *et al*. (2009).

#### Mating by minimising the covariance between ancestral contributions

Selected sires and dams were paired by minimising the sum of absolute values of the covariances between ancestral genetic contributions. The algorithm used to pair the sires and dams by MCAC with pedigree information is described in the Appendix of Henryon *et al*. (2009). We made one modification to the algorithm when MCAC with genomic information was used to pair sires and dams: matrices ***L*** and ***C*** were computed by decomposing the genomic-additive relationship matrix, ***G***, between ancestors and their descendants, where ***L*** is a normed lower triangular matrix describing the genetic contribution from ancestors to their descendants and ***C*** represents genetic contributions from the ancestors to allocated matings. Computation of ***C*** is presented in the Appendix (Supplementary Material S1).

#### Random mating

Selected sires and dams were paired randomly.

### Breeding schemes

The five breeding schemes are shown in Table 1, where scheme 1 is the hierarchical-breeding scheme that was used by Henryon *et al*. (2009). In total, 20 sires were selected in each breeding scheme. Each sire was mated to one (schemes 2 and 3), two (schemes 4 and 5) and six (scheme 1) dams. In schemes 1 and 2, 1200 offspring were generated in each generation; 400 offspring were generated in schemes 3 to 5. The heritability of the trait under selection was 0.1 in schemes 1 to 4. It was 0.4 in scheme 5.

**Table 1:** Details of the simulation of the five breeding schemes with different population structure (the number of selected dams and litter size) and heritability

Schemes	*N*_s_	*N*_d_	Litter size	Heritability
1	20	120	10	0.1
2	20	20	60	0.1
3	20	20	20	0.1
4	20	40	10	0.1
5	20	40	10	0.4

*N*_s_=number of selected sires. *N*_d_=number of selected dams.

### Simulation

**Figure 1:**
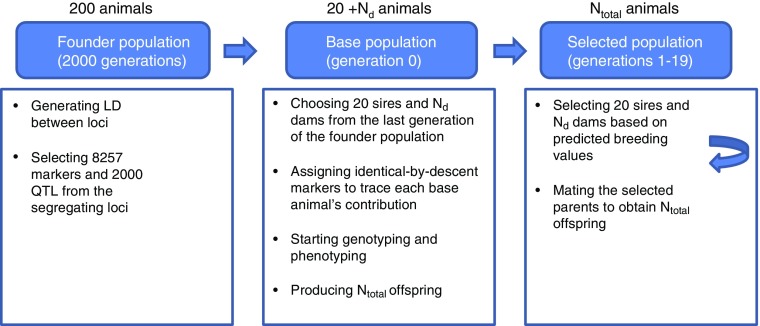
A summary of simulations. Simulations were carried out in the following three stages. In the first stage, 8257 markers and 2000 quantitative trait loci were generated by simulating a single founder population with a Fisher–Wright inheritance model. The founder population had an effective population size of 200 animals and 2000 generations, which was created to obtain desirable level of linkage disequilibrium between simulated loci. In the second stage, the base animals (in generation 0) were generated by choosing 20 sires and *N*_d_ dams from the last generation of the founder population. Two thousand identical-by-descent (IBD) markers were used to trace each base animal’s contribution to their descendant generations and infer IBD status relative the base population. In total, 20 sires and *N*_d_ dams in generation 0 were used to produce *N*_total_ offspring in generation 1. In the third stage, from generation 1 to 19, all *N*_total_ selection candidates were both genotyped and phenotyped before selection. In each generation, 20 sires and *N*_d_ dams were truncation-selected using breeding values predicted from a Ridge-Regression model and were mated to produce *N*_total_ offspring.

Simulations were carried out in three stages (Figure 1). In the first stage, we generated a single founder population. This founder population was used as the basis for the subsequent stages. In the second stage, we generated base population, which was run for 100 replicates. In the third stage, we generated selected population, which was run for 100 replicates.

#### Founder population and genetic architecture

The 8257 markers and 2000 QTL were generated by simulating a founder population with a Fisher–Wright inheritance model. The population had an effective population size of 200 animals and 2000 generations. Simulation of the genomes has been described in Liu *et al*. (2015). The simulated genome consisted of four 1 Morgan long chromosomes, on which 10 000 loci were equally distributed, resulting in 40 000 loci across the genome. The offspring inherited alleles at these loci from their parents following Mendel’s rules allowing for mutation and recombinations. Mutation was recurrent at a rate of 2.5×10^−5^ per locus per meiosis (Son *et al*., 2014; Liu *et al*., 2015). Recombinations per chromosome were sampled from a Poisson distribution with a mean equal to the length of the chromosome in Morgan and were randomly placed along the chromosome assuming a uniform distribution. In generation −1, the average (±SD) linkage disequilibrium (LD) between neighbouring loci was *R* ^2^=0.27 (±0.32), and the allele frequency distribution followed a U-shaped distribution, with ~30.2% of the loci fixed. Average *R* ^2^ between neighbouring loci was in the range of the estimation of LD in pig breeds (Badke *et al*., 2012).

Among all segregating loci, every second one with a minor allele frequency (MAF) >0.05 were used as markers. So in total, there were 8257 markers used for genomic prediction and mating strategies with genomic information in the simulated breeding schemes. Among the remaining segregating loci, 2000 loci with MAF>0.01 were used as QTL. The QTL allelic effects were assumed to follow a gamma distribution with a shape parameter of 1.48. This was the shape parameter derived for the distribution of QTL effects in pigs (Hayes and Goddard, 2001).

### Base population

We randomly sampled 20 sires and *N*_d_ dams from 200 animals in the last generation of the founder population, where *N*_d_ were the number of animals that were assigned to be sires and dams (Table 1). The base population produced the first generation of offspring (generation 1).

### Selected population

In generation 1 to 19, 20 sires and *N*_d_ were selected from *N*_total_ animals and mated to produce *N*_total_ offspring in each breeding scheme (Table 1). Offspring produced in generation 20 were the result of 19 generations of selection. The offspring in each generation inherited alleles at markers and QTL from their parents following Mendel’s rules allowing for recombinations. The simulation of recombination was the same as for the founder population.

### Tracking identity-by-descent

In total, 2000 identical-by-descent (IBD) markers were randomly distributed across the genome. These IBD markers were not involved in selection, but were assigned unique alleles to each base animal. They were used to trace each base animal’s contribution to their descendant generations and infer IBD status relative the base population. So within each locus of a descendant, each IBD marker allele could be traced directly back to the base animal from which it was derived. Any homozygous locus at IBD markers was an inbred locus. Inbreeding at each IBD marker was defined as the probability that two alleles at that locus from a randomly selected animal in the population are IBD.

### Phenotypes

The phenotype of the trait for the *i*th base animal, *y*_*i*_, was calculated as *y*_*i*_=*α*_*i*_+*e*_*i*_, where *α*_*i*_ is the base animal’s true additive-genetic value and *e*_*i*_ is its residual environmental value. The true additive-genetic value was calculated as the sum of 4000 QTL effects. The effects of those QTL were scaled to achieve an initial genetic variance equal to the heritability, that is 
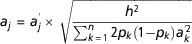
, where *h* ^2^ is 0.1 or 0.4, subscripts *k* (*j*) denote QTL *k* (*j*), *p*_*k*_ (*p*_*j*_) is the frequency of the ‘1’ allele of QTL *k* (*j*) and 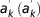 is the substitution effect of QTL *k* (*j*) before being scaled. The true breeding value for each animal was obtained by summing the allelic effects at each QTL. The additive QTL variance explained all additive-genetic variance 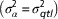. The environmental values were sampled from the distribution 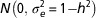. As a result, the phenotypes of the trait in the base population had a mean of 0 and a SD of 1. In descendant generations, QTL and tags were sampled according to principles of Mendelian inheritance. The environmental variance was constant through the simulation, such that genetic variance and heritability decreased over the course of generations of selection due to Bulmer-effect (Bulmer, 1971).

### Genomic prediction

Genomic-breeding values were predicted by fitting the ridge-regression model presented in Liu *et al*. (2015). The breeding value *g*_*i*_ for animal *i* was defined as a parametric linear regression on marker covariates *x*_*ij*_ of the form 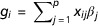, such that 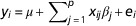, where *y*_*i*_ is the phenotypic record of an animal from generation *u* and *u*−1, *μ* is the intercept, *x*_*ij*_ takes the value 0, 1 or 2 for animal *i* and locus *j*, and 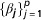 is a vector of marker effects (*j*=1, 2, …, *p* markers). Gaussian assumptions for model residuals were applied, that is the joint distribution of model residuals was assumed to follow 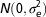. The likelihood function yields
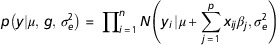
where 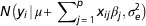 is a normal density for random variable *y*_*i*_ centered at 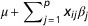 and with variance 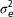. A common variance was assigned to all marker effects, that is 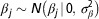. The predicted breeding value 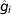 used for selection was defined as 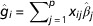.

### Assessment criteria

We present the rates of inbreeding and genetic gain realised by MC, MCAC and RAND with pedigree and genomic information. The inbreeding coefficient was calculated for each individual as the proportion of IBD markers that are homozygous. Rates of inbreeding, *∆F*, were calculated as 1−*e* ^*β*^, where *β* is slope of the linear regression of *ln*(1−*F*_*u*_) on *u* and *F*_*u*_ is the mean inbreeding coefficient for animals born at generation *u*, as used in Nirea *et al*. (2012). We did this transformation because theoretically the mean of inbreeding coefficients in a cohort becomes a linear function to the generation after data transformation, so that *∆F* is constant across the generations (Benoit, 2011). Genetic gain at generation *u* was calculated as the difference in average true breeding values between generation *u* and *u*−1 (1<*u*<20). We calculated *∆F* using *F*_*u*_ and rates of genetic gain (*∆G*) using true breeding values in generation 5 to 20. The difference between mating strategies with respect to Δ*F* and *∆G* were tested for significance using Tukey’s HSD (honest significant difference, *P*<0.05).

We also present findings that provide insight into the mechanisms that underlie any difference in Δ*F* or Δ*G* between mating strategies with genomic and pedigree information:

• The inbreeding coefficient (*F*_*u*_) over time.
• The genetic variance over time. The genetic variance was calculated as the variance of true breeding values in each generation.
• Genetic contributions.
  – The number of ancestors making a genetic contribution to the offspring in generation 20. We presented the average number of ancestors from generations 0 to 19 that made a genetic contribution to the offspring in generation 20 based on matrix ***C*** as described in Appendix (Supplementary Material S1) for genomic MCAC.
  – The deviation of long-term genetic contributions from the exact linear relationship between long-term genetic contributions and Mendelian-sampling terms. The Mendelian-sampling term was calculated as the difference between an animal’s true breeding value and the mean true breeding values of the parents of the animal. Long-term genetic contributions were computed based on both pedigree and genomic information (Henryon *et al*., 2009; Supplementary Material S1). Then the deviation was computed as the standard deviation of residuals from a linear regression of genetic contributions on Mendelian-sampling terms using the ancestors in generations 0 to 19 that made a genetic contribution to the offspring in generation 20.

All results are presented as means (and standard deviations across replicates) of the 100 simulation replicates.

## Results

### Rate of inbreeding

MC and MCAC with genomic information realised lower Δ*F* than MC and MCAC with pedigree information (Table 2). MC and MCAC with genomic information realised 6% to 22% less Δ*F* than with pedigree information in our five breeding schemes, which varied for mating ratio, litter size and heritability.

**Table 2:** Average rate of inbreeding (Δ*F*) realised by generation 5 to 20 in each of the pig breeding schemes

Schemes	Pedigree MC	Genomic MC	Pedigree MCAC	Genomic MCAC	RAND
1	0.053^bc^	0.050^bc^	0.055^b^	0.049^c^	0.072^a^
2	0.131^b^	0.108^c^	0.134^b^	0.113^c^	0.168^a^
3	0.093^b^	0.073^c^	0.087^b^	0.072^c^	0.128^a^
4	0.055^b^	0.048^c^	0.054^b^	0.049^c^	0.072^a^
5	0.054^bc^	0.050^c^	0.056^b^	0.051^c^	0.071^a^

Pedigree MC=minimum-coancestry mating with pedigree information; genomic MC=minimum coancestry mating with genomic information; pedigree MCAC=mating by minimising the covariance between ancestral genetic contributions with pedigree information; genomic MCAC=mating by minimising the covariance between ancestral genetic contributions with genomic information; RAND=random mating. The SD of means of 100 replicates of Δ*F* were <0.0044. ^a,b,c^Values within a row with different superscripts differ significantly (Tukey’s honest significant difference, *P*<0.05).

MC and MCAC using the same information source realised similar Δ*F* (Table 2). In turn, MC and MCAC with genomic and pedigree information realised 20% to 44% less Δ*F* than RAND.

The reduction in Δ*F* by using MC and MCAC with genomic information instead of pedigree information was larger when mating ratio, litter size and heritability were smaller in our breeding schemes. Mating ratio had the largest impact on the reduction in Δ*F*. The reduction in Δ*F* by using genomic information was 6% to 11% in scheme 1 with mating ratio of 6 dams/sire. It increased to 16% to 18% in scheme 2 with mating ratio of 1 dam/sire. Likewise, the reduction in Δ*F* was 9% to 13% in scheme 4 with mating ratio of 2 dams/sire. It increased to 17% to 22% in scheme 3 with mating ratio of 1 dam/sire. On the other hand, litter size and heritability had the smallest impact on the reduction in Δ*F*. The reduction in Δ*F* was increased from 16% to 18% to 17% to 22%, when the number of offspring per litter was decreased from 60 in scheme 2 to 20 in scheme 3. The reduction in Δ*F* was increased from 7% to 9% to 9% to 13%, when the heritability was decreased from 0.4 in scheme 5 to 0.1 in scheme 4.

### Rates of genetic gain

MC and MCAC with genomic and pedigree information realised similar Δ*G* within each of the five breeding schemes (Table 3). In turn, MC and MCAC with genomic and pedigree information realised 1% to 14% more Δ*G* than RAND.

**Table 3:** Average rate of genetic gain (Δ*G*) realised by different mating strategies at generation 5 to 20 in each of the pig breeding schemes

Schemes	Pedigree MC	Genomic MC	Pedigree MCAC	Genomic MCAC	RAND
1	0.155^ab^	0.156^ab^	0.158^ab^	0.159^a^	0.151^b^
2	0.145^bc^	0.150^ab^	0.141^cd^	0.154^a^	0.135^d^
3	0.129^a^	0.129^a^	0.127^a^	0.125^a^	0.120^b^
4	0.127^ab^	0.125^b^	0.126^ab^	0.131^a^	0.124^b^
5	0.325^a^	0.328^a^	0.327^a^	0.330^a^	0.314^b^

Pedigree MC=minimum-coancestry mating with pedigree information; genomic MC=minimum coancestry mating with genomic information; pedigree MCAC=mating by minimising the covariance between ancestral genetic contributions with pedigree information; genomic MCAC=mating by minimising the covariance between ancestral genetic contributions with genomic information; RAND=random mating. The SD of means of 100 replicates of Δ*G* were <0.001. ^a,b,c,d^Values within a row with different superscripts differ significantly (Tukey’s honest significant difference, *P*<0.05).

The following sections present findings that provide insight into the mechanisms that underlie the differences in Δ*F* between mating strategies with genomic and pedigree information.

### Inbreeding over time

**Figure 2:**
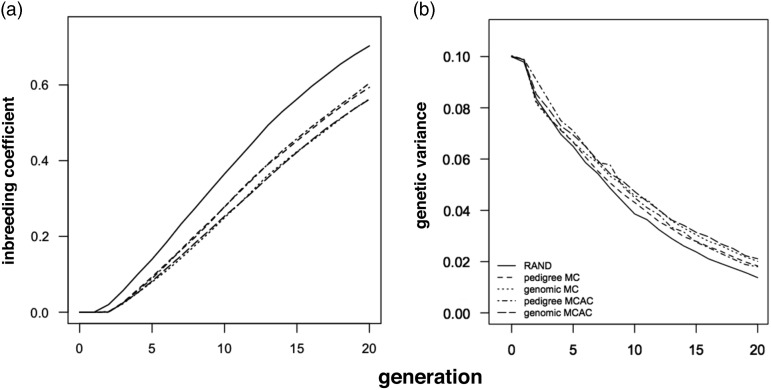
(a) Inbreeding coefficient and (b) genetic variance in each generation of selection in breeding scheme 1. Pedigree MC=minimum-coancestry mating with pedigree information; genomic MC=minimum coancestry mating with genomic information; pedigree MCAC=mating by minimising the covariance between ancestral genetic contributions with pedigree information; genomic MCAC=mating by minimising the covariance between ancestral genetic contributions with genomic information; RAND=random mating.

MC and MCAC with genomic information realised less inbreeding over time than MC and MCAC with pedigree information. This is illustrated for scheme 1 (Figure 2a). In this scheme, the onset of inbreeding with genomic and pedigree information was delayed until generation 3. After the onset of inbreeding, MC and MCAC with genomic information realised 6% to 11% less Δ*F* than with pedigree information. The result of delaying the onset of inbreeding and reducing Δ*F* with genomic information was that at generation 20, MC and MCAC with genomic information realised 3% to 8% less inbreeding than with pedigree information. In breeding schemes 2 to 5, MC and MCAC with genomic information realised 4% to 18% less inbreeding than with pedigree information at generation 20 (results not shown).

MC and MCAC with genomic and pedigree information realised less inbreeding over time than RAND. This was also illustrated for scheme 1, where the onset of inbreeding with RAND occurred one generation earlier than MC and MCAC with both genomic and pedigree information (generation 2 *v.* 3, Figure 2a). After the onset of inbreeding, MC and MCAC with genomic and pedigree information realised 24% to 32% less Δ*F* than RAND in scheme 1 (Table 2). In breeding schemes 2 to 5, MC and MCAC with genomic and pedigree information realised 19% to 41% less inbreeding than with RAND at generation 20 (results not shown).

### Genetic variance

MC and MCAC with genomic information maintained more genetic variance than with pedigree information. This is illustrated for scheme 1 (Figure 2b). The genetic variance maintained by MC and MCAC with genomic information (0.020) at generation 20 was 11% higher than with pedigree information (0.018). The variance maintained in RAND was only 0.014.

### Genetic contributions

MC and MCAC with genomic information had two impacts on realised genetic contributions of ancestors to their descendants. First, MC and MCAC with genomic information resulted in more ancestors making long-term genetic contributions to descendants in the populations than pedigree information (Table 4). With genomic information, the number of ancestors from generation 0 to 19 that made a genetic contribution to the offspring in generation 20 of each breeding scheme ranged from 11 to 47 per generation. The number of ancestors was 10 to 44 per generation with pedigree information. With RAND, the number of ancestors making contributions was only 8 to 41 per generation. Second, the genomic information reduced the standard deviation of residuals from the linear regression of genomic-based genetic contributions on Mendelian-sampling terms (Table 5). The standard deviation of residuals with genomic information ranged from 0.05 to 0.09 by using MC and MCAC at generation 20 in all breeding schemes. This was 2% to 7% less than with pedigree information. RAND generated highest standard deviation of residuals with genomic information, which ranged from 0.07 to 0.13 in all breeding schemes.

**Table 4:** Average number of ancestors in generations 0 to 19 that made a genetic contribution to offspring in generation 20 in all breeding schemes

Schemes	Pedigree MC	Genomic MC	Pedigree MCAC	Genomic MCAC	RAND
1	43.69	46.56	43.64	46.06	41.24
2	9.76	10.58	9.77	10.49	7.93
3	12.03	12.88	12.07	13.06	10.41
4	21.75	22.72	21.71	22.30	19.24
5	23.23	23.72	23.11	23.70	21.31

Pedigree MC=minimum-coancestry mating with pedigree information; genomic MC=minimum coancestry mating with genomic information; pedigree MCAC=mating by minimising the covariance between ancestral genetic contributions with pedigree information; genomic MCAC=mating by minimising the covariance between ancestral genetic contributions with genomic information; RAND=random mating. The SD of means of 100 replicates of the number of ancestors making a genetic contribution were <0.42.

**Table 5:** Mean of SD of residuals from a linear regression of genetic contributions on Mendelian-sampling terms for the ancestors in generations 0 to 19 that made a genetic contribution to the offspring in generation 20

	Pedigree MC		Genomic MC		Pedigree MCAC		Genomic MCAC		RAND	
Schemes	SD_ped_ ^1^	SD_gen_ ^2^	SD_ped_	SD_gen_	SD_ped_	SD_gen_	SD_ped_	SD_gen_	SD_ped_	SD_gen_
1	0.035	0.052	0.036	0.049	0.034	0.054	0.036	0.048	0.045	0.070
2	0.068	0.093	0.072	0.091	0.069	0.093	0.071	0.091	0.088	0.117
3	0.056	0.098	0.059	0.092	0.057	0.098	0.060	0.091	0.089	0.130
4	0.043	0.074	0.044	0.071	0.043	0.074	0.044	0.072	0.063	0.102
5	0.040	0.070	0.042	0.069	0.041	0.071	0.041	0.069	0.055	0.094

Pedigree MC=minimum-coancestry mating with pedigree information; genomic MC=minimum coancestry mating with genomic information; pedigree MCAC=mating by minimising the covariance between ancestral genetic contributions with pedigree information; genomic MCAC=mating by minimising the covariance between ancestral genetic contributions with genomic information; RAND=random mating. The SD of means of 100 replicates of SD_ped_ were <0.0024 and those of SD_gen_ were <0.0041.

1 The deviation of long-term genetic contributions from exact linear relationship with true Mendelian-sampling terms. It was achieved by presenting the SD of residuals from the linear regression of pedigree-based genetic contributions on Mendelian-sampling terms.

2 The deviation of long-term genetic contributions from exact linear relationship with true Mendelian-sampling terms. It was achieved by presenting the SD of residuals from the linear regression of genomic-based genetic contributions on Mendelian-sampling terms.

## Discussion

Our results supported the hypothesis that MC and MCAC with genomic information realise lower rates of inbreeding than MC and MCAC with pedigree information without compromising rates of genetic gain. The 6% to 22% reduction in Δ*F* by using genomic information with these mating strategies was realised over a range of breeding schemes using truncation selection. These reductions are worthwhile in practical breeding schemes because they can be achieved without extra costs and logistical constraints. All of the information required to implement MC and MCAC with genomic information is available from genomic prediction, which was the initial reason for implementing genomic information in breeding schemes. The mating strategies use genomic information to pair the parents more appropriately. They disperse genetic contributions of ancestors more widely, allowing selection to align the ancestors closer to the exact threshold-linear relationship. This is clearly an improvement on pedigree information. Therefore, genomic information should be applied to more than just prediction of breeding values in breeding schemes with truncation selection.

As we proposed, MC and MCAC with genomic information realised lower Δ*F* than with pedigree information for two reasons. First, MC and MCAC with genomic information increased independence and reduced confounding between the genetic contributions of ancestors. Second, this increased independence enabled selection to increase the contributions of ancestors with the largest Mendelian-sampling terms. The contributions of ancestors stabilised closer to the exact linear relationship between the long-term genetic contributions and Mendelian-sampling terms of the ancestors. It resulted in more ancestors making long-term contributions to descendant animals. We verified this with our analyses of genetic contributions. This underlying mechanism, linking truncation selection to genetic gain and inbreeding applies across a broad range of breeding schemes, species and genetic architectures. So, we recommend that MC or MCAC with genomic information become the method-of-choice for any breeding scheme with truncation selection because it should always realise lower Δ*F*.

Although we expect these mechanisms to apply to any breeding scheme, species and genetic architecture, the reduction in Δ*F* by using genomic information was largest with small mating ratio, litter size and heritability in our breeding schemes. Mating ratio had the largest impact on the reduction in Δ*F* because it affects the independence of genetic contributions of ancestors. With small mating ratio, there was more dependence between contributions of sires and dams to be broken up by MC and MCAC. In contrast, there was less dependence between the contributions with large mating ratio, and therefore, there was less to be gained by using MC and MCAC with genomic information. On the other hand, litter size and heritability had the smallest impact on the reduction in Δ*F*. With small litter size, dependence between contributions of sires and dams were larger, because there were fewer reshuffled chromosome segments inherited from the parents to the offspring. In contrast, with large litter size, many recombinations occurred and more combinations of chromosome segments were created in the offspring. Then genomic selection can select animals carrying diverse combinations of chromosome segments to mate subsequently, allowing for new degrees of freedom to be generated for allocating mates. In this case, there was less to be gained by using genomic information compared with small litter size. With low heritability, the selection was less accurate, so that more independence was required for subsequent changing of genetic contributions. More independence can be achieved by using genomic information. Therefore, although MC and MCAC with genomic information should always realise lower Δ*F*, we will gain most benefit by implementing MC and MCAC with genomic information in breeding schemes when there is less independence between genetic contributions of ancestors or where more independence is required.

Not only did MC and MCAC with genomic information reduce Δ*F*, they did so without compromising genetic gain for two reasons. First, MC and MCAC with genomic information reduced the deviations of genetic contributions from the threshold-linear relationship without changing the slope of the relationship. It resulted in the same selection intensity and the same accuracy realised by MC and MCAC with pedigree and genomic information. Second, we are able to maintain larger genetic variance by using MC and MCAC with genomic information, so we can obtain as much genetic gain as using MC and MCAC with pedigree information (Woolliams *et al*., 1999). Therefore, by applying MC and MCAC with genomic information, we are able to guarantee a breeding programme with less inbreeding without sacrificing any benefit.

Although mating with genomic information can better manage Δ*F* than with pedigree information, it was surprising to find similar Δ*F* and Δ*G* realised by MC and MCAC, even though they use different criteria to allocate mates. MC improves the family structure by minimising the variance of ancestral genetic contributions for a randomly chosen offspring, whereas MCAC minimises the covariance of ancestral genetic contributions (Caballero *et al*., 1996; Sonesson and Meuwissen, 2000; Henryon *et al*., 2009; Nirea *et al*., 2012). The reason that the differences in Δ*F* between MC and MCAC were small was that the mating strategy that controls the variance of genetic contributions also controls covariance between genetic contributions (Supplementary Tables S1 and S2 and Supplementary Figure S1). This means that the mechanisms underlying MC and MCAC are similar even though these two mating strategies applied different methodologies to achieve lower Δ*F*. Therefore, it does not matter which one to use in practical breeding schemes with truncation selection and genomic information.

There are numerous ways to estimate realised coancestry and genetic contributions directly from the marker data (Speed and Balding, 2015). The ***G*** matrix we estimated was genome-wise averages of single-SNP statistics, which was similar to VanRaden’s ***G*** matrices in that it did not take lengths of the genomic regions shared between individuals into account (VanRaden, 2008). Edwards (2015) proposed measures of coancestry based on models for the joint distribution of markers at the haplotype level. These measures can estimate genealogical relatedness more accurately than vanRaden’s ***G*** particularly for distantly related individuals. The use of haplotype methods can track the way the alleles inherited from parents to offspring more accurately, which may further increase the independence between the contributions of sires and dams compared with ***G*** matrices. If this is the case, then we would expect that MC and MCAC with relationship matrices estimated using haplotype methods will generate even lower rates of inbreeding than using ***G*** matrices.

## Supplementary material

For supplementary material accompanying this paper visit http://dx.doi.org/10.1017/S1751731116001786. click here to view supplementary material
