# Supplementary material for: Mating strategies with genomic information reduce rates of inbreeding in animal breeding schemes without compromising genetic gain
Source: Animal. 2016 Aug 17;11(4):547–55. doi: 10.1017/S1751731116001786 (PMC5361395; doi:10.1017/S1751731116001786)
Supplement: Supplementary file 1 [file S1751731116001786sup001.docx]

Mating strategies with genomic information reduce rates of inbreeding in animal breeding schemes without compromising genetic gain

H. Liu^1^, M. Henryon^2,3^, A.C. Sørensen^1^

^1^Center for Quantitative Genetics and Genomics, Department of Molecular Biology and Genetics, Aarhus University, P. O. Box 50, 8830 Tjele, Denmark

^2^Seges, Danish Pig Research Centre, Axeltorv 3, 1609 Copenhagen V, Denmark

^3^School of Animal Biology, University of Western Australia, 35 Stirling Highway, Crawley, WA 6009, Australia

Supplementary Material S1

Appendix

The process of computing genetic contribution matrix$\boldsymbol{C}$ in genomic MCAC was as follows:

1. Initialize the variable$t=0$, where $t$ is the generation where ancestors come from.
2. Construct $\boldsymbol{M}_{u,t}$ matrix, which is an ${(a}_{t}+d_{u})\times n$genotypic matrix, where$u$ is the generation where selected parents for mating are from ($u>t$), $n$ is the number of markers, $a_{t}$is equal to the total number of animals from the generation $t$ and $d_{u}$ from generation u.
3. Construct genomic relationship $\boldsymbol{G}$ matrix, which is an ${(a}_{t}+d_{u})\times{(a}_{t}+d_{u})$matrix according to Yang et al. 2010 , given $\boldsymbol{M}_{u,t}$ matrix and the allele frequencies of markers in the base population$\boldsymbol{p}_{0}$.
4. Construct additive relationship $\boldsymbol{A}$ matrix, which is an ${(a}_{t}+d_{u})\times{(a}_{t}+d_{u})$matrix computed based on the full pedigree. To make $\boldsymbol{G}$ a positive definite matrix and a matrix that is on the same scale as $\boldsymbol{A}$ matrix, $\boldsymbol{G}$ matrix was adjusted for the difference between the original genomic relationship matrix and $\boldsymbol{A}$. The $\boldsymbol{G}$ matrix was adjusted using two parameters $\alpha$ and $\beta$, i.e.

$$\boldsymbol{G}^{\boldsymbol{'}}=\boldsymbol{G}\beta+\alpha$$

which were derived from the following equations:

$$Average of diag\left( \boldsymbol{G} \right)\beta+\alpha=Average of diag(\boldsymbol{A}_{11})$$

$$Average of offdiag\left( \boldsymbol{G} \right)\beta+\alpha=Average of offdiag(\boldsymbol{A}_{11})$$

Then the $\boldsymbol{G}$ matrix was adjusted by combining matrices $\boldsymbol{A}$ and $\boldsymbol{G}$:

$$\boldsymbol{G}^{*}=\omega\boldsymbol{A}+\left( 1-\omega\right)\boldsymbol{G}^{'}$$

where $\omega$ was set to 0.2.

$\boldsymbol{G}^{*}$ was decomposed to a normed lower-triangular matrix $\boldsymbol{L}_{u,t}$. From $\boldsymbol{L}_{u,t}$ we extract $\boldsymbol{C}_{u,t}$, which is a $d_{u}{\times a}_{t}$matrix of contribution from ancestors at generation $t$ to descendants at generation u.

1. Construct an $m\times a_{t}$matrix $\boldsymbol{C}_{u+1,t}$, where $m$ is the number of matings for generation $u+1$. Element $\boldsymbol{C}_{u+1,t (j,k)}$ represents the genetic contribution of the $k$th ancestor to the offspring of the $j$th mating, which was obtained from the equation:

$\boldsymbol{C}_{u+1,t (j,k)}={\frac{1}{2}\boldsymbol{C}}_{u, t \left( sire\left( j \right),k \right)}+ {\frac{1}{2}\boldsymbol{C}}_{u, t \left( dam\left( j \right),k \right)}$, where $sire\left( j \right)$ and $dam\left( j \right)$ represents the sire and dam of $j$th mating. Based on the pedigree, if individual $j$ is not a descendant of individual$k$, $\boldsymbol{C}_{u+1,t (j,k)}$ is set to 0. Then $\boldsymbol{C}_{u+1,t}$ under $t=0$was recorded.

1. Set $t=t+1$.
2. Repeat steps 1-6 until $t=u-1$.
3. Combine all $\boldsymbol{C}_{u+1,t}$ computed based on $t=0, ... u-1$ by columns. The combined matrix was denoted as $\boldsymbol{C'}$**,** which is a $m\times\left( a_{0}+a_{1}\ldots+a_{u-1} \right)$ matrix.

Now $t=u$, then

$\boldsymbol{C}_{u+1,t (j,k)}=\frac{1}{2}$, if $k$ is the sire or the dam of $j$.

$\boldsymbol{C}_{u+1,t (j,k)}=0$, if $k$ is a parent of $j$.

1. Combine $\boldsymbol{C'}$ and $\boldsymbol{C}_{u+1,t (j,k)}$ under $t=u$ by columns. The combined matrix was denoted as $\boldsymbol{C}$**,** which is a $m\times\left( a_{0}+a_{1}\ldots+a_{u} \right)$ matrix**_._**

**Reference**

Yang J, Benyamin B, McEvoy BP, Gordon S, Henders AK, Nyholt DR, Madden PA, Heath AC, Martin NG, Montgomery GW et al 2010: Common SNPs explain a large proportion of the heritability for human height. Nature Genetics 42, 565-569.

Supplementary Material S2

**Analyses of mechanisms underlying different mating strategies**

The non-zero true genetic contribution from a base animal to a descendant was calculated as the proportion of IBD marker alleles contributed from this base animal. To obtain the variance of genetic contributions for each mating strategy, the variance of the non-zero true genetic contributions of base animals to each of the descendants were calculated and these variances were averaged over descendants and replicates. To obtain covariance between genetic contributions of all base animals for each mating strategy, the co-variances of non-zero true genetic contributions between base animals to all their descendants were summed and averaged over replicates.

**Supplementary Table S1** *The average of variance of genetic contributions per offspring and the average of the sum of covariance of ancestral contributions over all of the offspring based on 20 replicates*

|  | pedigree MC | genomic MC | pedigree MCAC | genomic MCAC | RAND |
| --- | --- | --- | --- | --- | --- |
| VAR | 0.0062 | 0.0054 | 0.0076 | 0.0055 | 0.0117 |
| COV | 0.0350 | 0.0311 | 0.0326 | 0.0308 | 0.0437 |

pedigree MC = minimum-coancestry mating using pedigree information;

genomic MC = minimum coancestry mating using genomic information;

pedigree MCAC = mating by minimizing the covariance between ancestral genetic contributions using pedigree information;

genomic MCAC = mating by minimizing the covariance between ancestral genetic contributions using genomic information.

RAND = random mating.

The breeding scheme was a modification of breeding scheme 2.

The variance of genetic contributions was smaller with pedigree MC than pedigree MCAC, while covariance between genetic contributions was smaller with pedigree MCAC than pedigree MC (Supplementary Table S1). However, both the variance and covariance of genetic contributions were virtually identical between genomic MC and MCAC. RAND caused the largest variance and covariance of genetic contributions.

We also calculated the correlation between $\Delta F$ in the entire population and variance or covariance of genetic contributions realised by MC and MCAC and RAND to understand which one is more associated with $\Delta F$.

**Supplementary Table S2** *The correlation between the rate of inbreeding (*$\Delta F$*) and the variance of genetic contributions or covariance between genetic contributions.*

|  | pedigree MC | genomic MC | pedigree MCAC | genomic MCAC | RAND |
| --- | --- | --- | --- | --- | --- |
| COR_VAR_ | 0.448 | 0.824 | 0.656 | 0.605 | 0.652 |
| COR_COV_ | 0.348 | 0.610 | 0.358 | 0.485 | 0.393 |

pedigree MC = minimum-coancestry mating using pedigree information;

genomic MC = minimum coancestry mating using genomic information;

pedigree MCAC = mating by minimizing the covariance between ancestral genetic contributions using pedigree information;

genomic MCAC = mating by minimizing the covariance between ancestral genetic contributions using genomic information.

RAND = random mating.

COR_VAR_= The correlation between the rate of inbreeding ($\Delta F$) and the variance of genetic contributions

COR_COV_= The correlation between the rate of inbreeding ($\Delta F$) and covariance between genetic contributions.


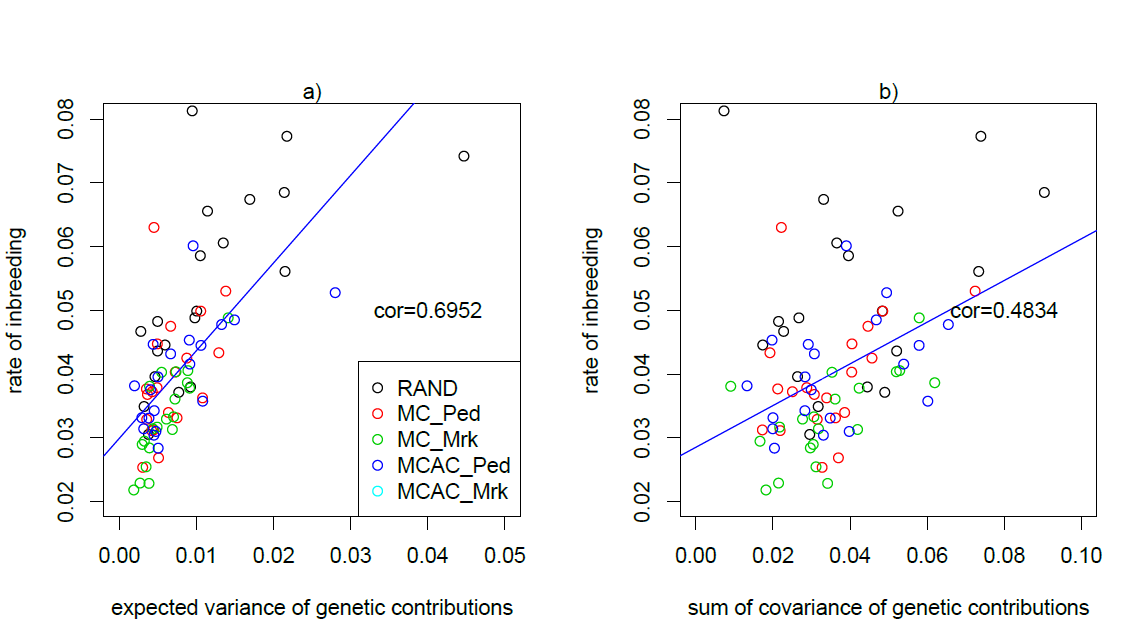


**Supplementary Figure S1**: *a) the rate of inbreeding was plotted against the variance of genetic contributions per offspring and the sum of covariance of ancestral contributions over all of the offspring for each mating strategy and for each replicate.*

In general, the correlation between $\Delta F$ and variance of genetic contributions was higher than the correlation between $\Delta F$ and the covariance between genetic contributions. The former ranged from 0.448-0.823 and the latter ranged from 0.348-0.610 (Supplementary Table S2). Among all these mating strategies, genomic MC caused highest correlations. It shows that the correlation between $\Delta F$ and variance of genetic contributions was higher (~0.70) than the correlation between $\Delta F$ and covariance between genetic contributions (~0.48) when the results from all mating strategies were pooled (Supplementary Figure S1).
